# Viruses and amyloids - a vicious liaison

**DOI:** 10.1080/19336896.2023.2194212

**Published:** 2023-03-30

**Authors:** Per Hammarström, Sofie Nyström

**Affiliations:** IFM-Chemistry, Linköping University, Linköping, Sweden

**Keywords:** Amyloid, long COVID, PASC, post-acute sequalae COVID-19, SARS-CoV-2, virus

## Abstract

The crosstalk between viral infections, amyloid formation and neurodegeneration has been discussed with varying intensity since the last century. Several viral proteins are known to be amyloidogenic. Post-acute sequalae (PAS) of viral infections is known for several viruses. SARS-CoV-2 and COVID-19 implicate connections between amyloid formation and severe outcomes in the acute infection, PAS and neurodegenerative diseases. Is the amyloid connection causation or just correlation? In this review we highlight several aspects where amyloids and viruses meet. The evolutionary driving forces that dictate protein amyloid formation propensity are different for viruses compared to prokaryotes and eukaryotes, while posttranslational endoproteolysis appears to be a common mechanism leading up to amyloid formation for both viral and human proteins. Not only do human and viral proteins form amyloid irrespective of each other but there are also several examples of co-operativity between amyloids, viruses and the inter-, and intra-host spread of the respective entity. Abnormal blood clotting in severe and long COVID and as a side effect in some vaccine recipients has been connected to amyloid formation of both the human fibrin and the viral Spike-protein. We conclude that there are many intersects between viruses and amyloids and, consequently, amyloid and virus research need to join forces here. We emphasize the need to accelerate development and implementation in clinical practice of antiviral drugs to preclude PAS and downstream neurological damage. There is also an ample need for retake on suitable antigen targets for the further development of next generation of vaccines against the current and coming pandemics.

## Introduction

Viral infections are prevalent in humans and in an absolute majority of cases the consequent disease is mild and disease outcome is good. However, viral infections can potentially drive long-term disease [1] and in particular increase the risk of common degenerative diseases such as Alzheimer’s and Parkinson’s disease [2] associated with protein misfolding and amyloidosis. In this review we provide an overview of viral infections and viral proteins and molecular overlaps with amyloid diseases and amyloid proteins in humans. Corona viruses have been circulating among humans for a long time, probably for centuries but were first identified, isolated and classified in the 1960s. The common corona viruses (HCoV-NL63, HCoV-229E, HCoV-OC43 and HKU1) give rise to mild respiratory symptoms and, in the case of HKU1, gastrointestinal symptoms have been noted [3]. These viruses are in constant circulation in the human population, yet severe infection is extremely rare in immune responsive adults. More recently two novel corona viruses, SARS-CoV (years 2002–2003) and MERS-CoV (year 2012) have threatened to cause pandemics but were kept under control. Both these diseases were in the majority of cases respiratory diseases albeit some MERS patients also presented with gastrointestinal symptoms [4]. The novel corona virus, SARS-CoV-2, hit the world hard starting late2019. In March 2020 WHO declared that COVID-19 was to be characterized as a pandemic. In the early days of the disease outbreak, COVID-19 was portrayed as respiratory disease with many commonalities with previous outbreaks of SARS and MERS. However, it soon became evident that this novel disease hit multiple organ systems throughout the body [5] and in an autopsy study of German patients from the first months of the pandemic it was concluded that majority of the 26 studied patients also suffered from multiorgan failure [6], often connected to thromboembolism [7] and hyperinflammation [7].

COVID-19 is far from only a respiratory disease [8]. Although the most common site of virus entry is from the respiratory tract, there is evidence for virus replication in several other tissue types: renal, myocardial, neurological, pharyngal and gastrointestinal [9]. ACE2, the receptor for viral entry into host cells, is abundantly expressed in several tissues suggesting a route of direct toxic exertion of the virus onpancreatic insulin producing β-cells [10]. Systemic involvement such as immune response dysregulation (hyperinflammation and cytokine storms), haematological abnormalities (lymphopenia, neutrophilia, thrombocytopenia) and endothelial damage, leading up to immunothrombosis is also a common non-respiratory manifestation of SARS-CoV-2 infection [8]. Cardiac complications such as ischaemic myocardial injuries, arrythmias, and cardiac arrest are also noted as severe outcomes of COVID-19 infection [11]. Neurological symptoms such as anosmia and ageusia were early in the pandemic recognized as common and specific traits of COVID-19 regardless of severity of disease. Reviewing hospitalized patients for any neurological symptom one year into the pandemic revealed that one third experienced at least one of in total 24 neurological symptom described. Looking at symptoms stemming from the central nervous system shows that around one third of patients experienced fatigue and of age above 60 one third exhibited delirium and/or confusion [12]. Of the patients in the study, 1 in 50 hospitalized patients suffered a stroke. Renal, hepatic and endocrine injuries are frequently reported in association to COVID-19 hospitalization. A New York study showed that 36% of hospitalized patients during March 2020 developed acute kidney injury with the number elevated to 89% when looking only at patient requiring mechanical ventilation [13]. A meta-analysis looking for hepatic complications found abnormal liver function in 19% of COVID-19 patients with a clear increase in severe cases [14]. Diabetes is associated to higher risk of severe COVID-19. However, COVID-19 also imposes a risk of insulin resistance [15] normally associated to type 2 diabetes. In addition, many of the endocrine glands express the ACE2 receptor as well as the protease TMPRSS2 that enables viral entry to the cell, making them a target for direct viral infection [16]. Among other, both the thyroid and both male and female reproductive glands have been reported to be affected during SARS-CoV-2 infection [17].

## Post-acute sequalae (PAS) as result of viral infection

Viral infection can cause permanent injury in the host to various degrees and by different mechanisms. As examples can be mentioned Herpes viruses that inflict chronic infection on the host by incorporating its DNA into the chromosomal DNA in the nuclei of the host cell [18]. It will hence be dormant in the infected cell and ancestral cells. Other viruses, such as Polio [19] and Rubella [20], can during the acute infection cause irreparable and debilitating damage.

Post-acute sequalae (PAS), on the contrary, is a term used for symptoms that occur for the first time after the acute infection is healed and virus particles no longer can be detected by PCR [1]. PAS has been described for a large number of viruses, such as Ebola, Epstein-Barr virus, Zika virus, tick-borne encephalitis virus, and possibly also influenza (for comprehensive lists see [1] and [21]). PAS has also been reported for SARS and MERS but notably not been described for any of the four above mentioned common corona viruses (HCoV-NL63, HCoV-229E, HCoV-OC43 and HKU1).

Previous corona pandemics SARS and MERS have been connected to PAS and a recent review of these state that 20–30% of those hospitalized with SARS or MERS were not back to full working capacity one year after admission from hospital [22]. Looking closer at long-term effects of SARS and MERS demonstrate that a large portion of survivors exhibit pulmonary abnormalities that persist after the acute infection has diminished. Chest X-ray follow up 32–230 days after recovery showed that 38% of MERS patients exhibited lung fibrosis and/or pleural thickening [23]. Of Hong Kong university hospital health care workers affected by SARS (mean age 36 years), 24% had decreased lung capacity 1 year after recovery and 28% had abnormal chest X-rays [24]. Long-term follow-up of a different SARS patient cohort (health care workers from Peking general hospital) reveal that the vast majority of patients fully recover during the following one to two years [25]. However, this follow-up study of healthcare workers that were infected by SARS at Peking general hospital in 2002 demonstrates that around 5% still have interstitial pulmonary changes detectable in CT scan after 15 years [25].

Mental health disturbances and chronic fatigue syndrome were followed up among SARS patients in Hong Kong 4 years after recovery, and 40% of the patients were reported to have active psychiatric illness. The CDC criterion for chronic fatigue syndrome was fulfilled for 27% of the patient group [26]. Similar results were reported in a 12 months follow-up of MERS patients in South Korea where around 40% of patients were reported to show symptoms of Chronic fatigue syndrome while depression and PTSD were reported in 20–30% of survivors of MERS [27].

### Long COVID/PASC

**Figure 1.**
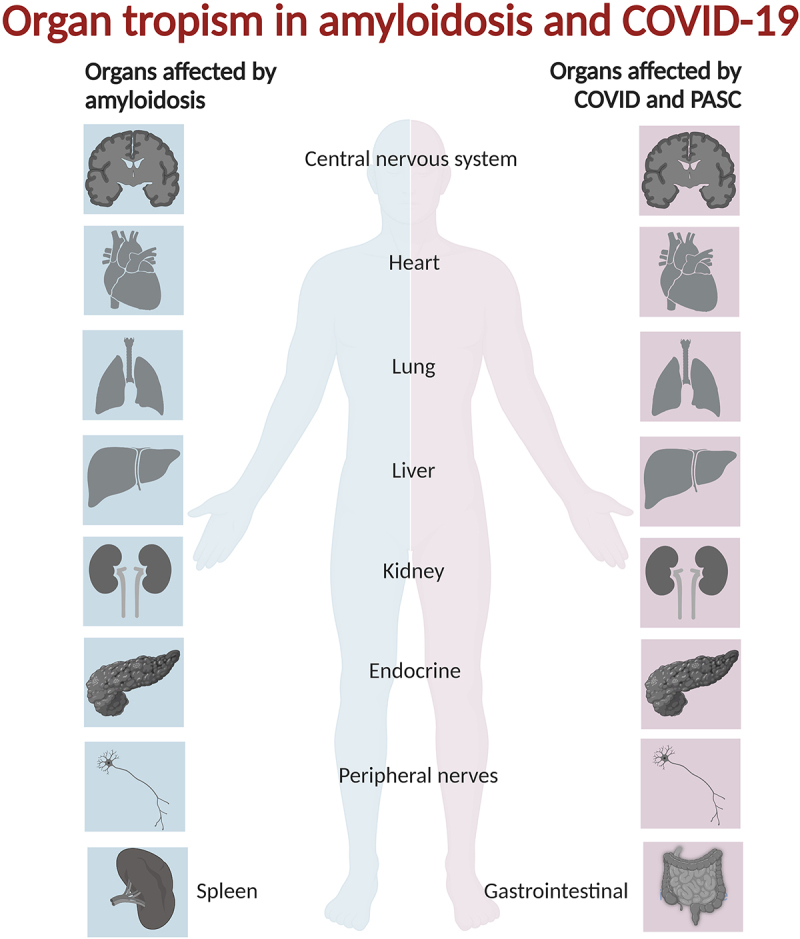
Organs and tissues affected by systemic and localized amyloidosis are in many instances overlapping with those afflicted by acute COVID-19 and PASC.

The nomenclature for PAS after infection with SARS-CoV-2 is not standardized but WHO has defined the condition as ‘a condition that occurs in individuals with a history of probable or confirmed SARS-CoV-2 infection, usually 3 months from the onset of COVID-19 with symptoms that last for at least 2 months and cannot be explained by an alternative diagnosis’. This condition is often referred to as long COVID or PASC. Over half of patients (56%) that have been infected with SARS-CoV-2 have at least one symptom of long covid at 6 month follow-up and the outcome cannot be correlated to severity of the acute disease [28]. Both neurological and psychiatric symptoms, lung abnormalities and chronic fatigue have been reported as PAS of COVID-19 (PASC). However, the list of symptoms exceeding this is extensive. Neurological, psychiatric, fatigue and respiratory symptoms each are reported in approximately 20% of cases, on par with SARS and MERS. In addition, a plethora of symptoms from the digestive tract and cardiovascular system as well as movement disabilities, hair and skin lesions are present in 6–17% of cases (Figure 1).

## The amyloid connection – causation or just correlation?

Protein processing, misfolding, and aggregation cause degenerative amyloid diseases. These diseases can manifest in most organs of the body and are dependent on the aggregating amyloid protein being specific for each disease (Table 1). Listing organs involved in amyloid diseases [29] side by side with those affected by COVID-19 [5] and PASC [30] it is easy to see there are many commonalities (Figure 1). But correlation is not always causation. We therefore decided to look closer at molecular associations and pathways of both amyloid proteins encoded by the human genome and amyloid forming proteins of viral origin.

**Table 1.** Human amyloid fibril proteins and their precursors. Table is modified from [29]. Proteins are listed in groups as full-length protein in fibril, fragments in fibril, and endoproteolytic products from precursor proteins.

Fibril protein	Precursor protein	Tropism	Target organs	Full length functional protein in fibril	Fragments of functional protein in fibril	Endo-proteolytic processing required for amyloidogenesis*
Aβ2 M	β2-microglobulin, wild type	Systemic	WT: Musculoskeletal system; Mut: ANS, tongue, heart	X		
AApoAII	Apolipoprotein A II, variants	Systemic	Kidney	X		
AApoCII	Apolipoprotein C II, variants	Systemic	Kidney	X		
AApoCIII	Apolipoprotein C III, variants	Systemic	Kidney	X		
ALECT2	Leukocyte chemotactic factor-2	Systemic	Kidney, primarily	X		
ACys	Cystatin C, variants	Neurologic	CNS, skin	X		
ALac	Lactoferrin	Localized	Cornea	X		
AL	Immunoglobulin light chain	Systemic	All organs, usually except CNS	X	X	
AH	Immunoglobulin heavy chain	Systemic	All organs except CNS	X	X	
ATTR	Transthyretin, wild type and variants	Systemic	WT: Heart mainly in males, lung, ligaments, tenosynovium; Mut:PNS, ANS, heart, eye, kidneys, leptomeninges	X	X	
AApoAI	Apolipoprotein A I, variants	Systemic	Heart, liver, kidney, PNS, testis, larynx (C-terminal variants), skin (C-terminal variants)	X	X	
ALys	Lysozyme, variants	Systemic	Kidney, Gastrointestinal	X	X	
AαSyn	α-Synuclein wild type and variants	Neurologic	CNS, ANS	X	X	
ATau	Tau wild type and variants	Neurologic	CNS	X	X	
APrP	Prion protein, wild type and variants	Neurologic	WT: CJD, fatal insomnia; Mut: CJD, GSS syndrome, fatal insomnia, PNS	X	X	
AKer	Kerato-epithelin TGFB1	Localized	Cornea, hereditary	X	X	
AA	(Apo) Serum amyloid A	Systemic	All organs except CNS		X	X
AApoAIV	Apolipoprotein A IV, wild type	Systemic	Kidney medulla and systemic		X	X
AGel	Gelsolin, variants	Systemic	PNS, cornea, Kidney		X	X
AFib	Fibrinogen, α, variants	Systemic	Kidney, primarily		X	X
ABri	ABriPP, variants	Neurologic	CNS		X	X
ADanb	ADanPP, variants	Neurologic	CNS		X	X
Aβ	Aβ protein precursor, wild type and variants	Neurologic	CNS		X	X
ATMEM106B	Transmembrane 106B (TMEM106B)	Neurologic	CNS		X	X
AMed	Lactadherin (MFG-E8)	Localized	Ageing aorta, media, elastic arteries		X	X
ACal	(Pro)calcitonin	Localized	C-cell thyroid tumours, Kidney			X
AIAPP	Islet amyloid polypeptide	Localized	Islets of Langerhans, insulinomas			X
AANP	Atrial natriuretic peptide	Localized	Cardiac atria			X
APro	Prolactin	Localized	Pituitary prolactinomas, ageing pituitary			X
ASom	(Pro)somatostatin	Localized	Somatostatinomas			X
AGluc	Glucagon	Localized	Glucagonomas			X
APTH	Parathyroid hormone	Localized	Parathyroid tumours, Ageing parathyroid glands			X
ASPC	Lung surfactant protein	Localized	Lung			X
ACor	Corneodesmosin	Localized	Cornified epithelia, hair follicles			X
AOAAP	Odontogenic ameloblast-associated protein	Localized	Odontogenic tumours			X
ASem1	Semenogelin 1	Localized	Vesicula seminalis			X
ACatK	Cathepsin K	Localized	Tumour associated			X
AEFEMP1	EGF-containing fibulin-like extracellular matrix protein 1 (EFEMP1)	Localized	Veins, Aging associated			X
Ains**	Insulin	Localized	Iatrogenic, local injection			X**
Aenf**	Enfurvitide	Localized	Iatrogenic, local injection			X**
AGLP1**	Glucagon-like peptide 1 analog	Localized	Iatrogenic, local injection			X**
AIL1RAP**	Interleukin-1 receptor antagonist protein	Localized	Iatrogenic, local injection			X**

*not including signal peptide processing.

**iatrogenic provided by injection of medical products.

### Proteins that cause amyloid diseases in humans

In humans 42 proteins are classified as amyloid proteins or protein precursors of amyloid fibrils [29] (Table 1 and examples below). In addition, some of these proteins are also associated with intracellular aggregates and there are proteins which appear to only form intracellular inclusions upon aggregation [29]. It is notable that the amyloid proteins in human disease are of highly diverse sequence, fold, and function. The molecular initiation events and driving force for amyloidosis appear to be rather different for different diseases and proteins.

There are also mammalian and more specifically also human endogenous proteins where amyloid formation plays a vital functional role [31]. Storage of peptide hormones in secretory granules [32] and Zona Pellucida protein of oocytes that form amyloid which play several vital roles in the early stages of fertilization [33] are two representative examples.

### Systemic amyloidosis

**AA –** AA amyloidosis or secondary amyloidosis is caused by chronic inflammatory diseases such as lupus, Sjogren’s syndrome, and Rheumatoid arthritis. These chronic inflammatory diseases render 1000-fold higher expression of the acute phase hepatic SAA1 protein than under normal conditions [34,35]. Hereditary familial Mediterranean fever (FMF), occurring due to mutations in the MEFV (pyrin) gene, results in lower expression of active IL-1Beta. This is suggested to result in elevated responsiveness to proinflammatory interleukins, mainly IL-1 sensitivity rendering IL-6 upregulation and thereby overexpression of SAA1 acute phase activation protein [36]. As a secondary consequence of this chronic inflammation an amyloid fibril seed forms by continuous SAA1 overexpression and proteolytic processing thereof, with can progress to AA amyloidosis. AA amyloidosis predominantly affects the kidney and can progress to a systemic amyloidosis in several organs including the heart [37].

**AFib-** Fibrinogen, the precursor of blood clot response protein, also employs extracellular protein fibre formation as a vital part of haemostasis and to enable wound healing [38]. Fibrinogen is composed of three separate protein subunits making heterotrimers that dimerize into a hexameric oligomer. Fibrinogen is synthesized in the liver at a very high rate and is secreted to circulation. Fibrinogen is an amyloidogenic protein. Familial amyloid disease that originates from point mutations in fibrinogen alpha-chain renders aggregation prone folding intermediates or degradation intermediates [39]. Mutant protein is however not found in circulation suggesting a rapid accumulation of the alpha-subunit as AFib amyloid in the kidney [40]. Frame shift mutations render a truncated form with a common amino acid sequence that forms amyloids [41]. The fibrinogen alpha-chain peptide 148–160 (KRLEVDIDIKIRS) formed amyloid fibrils whereas the shorter 148–157 peptide did not [42]. Notably no wild type fibrinogen is found deposited within familial amyloid deposits of AFib.

**ATTR –** Transthyretin (TTR) is a plasma protein synthesized mainly in the liver and the choroid plexus. TTR is a transport protein of thyroxine and retinol in blood plasma and cerebrospinal fluid. Transthyretin amyloidosis of wild type TTR can cause cardiomyopathy (ATTR-CM) in elderly patients and hereditary forms of TTR amyloidosis such as familial amyloid polyneuropathy (FAP) at younger ages [43]. ATTR fibrils accumulate and encircle normal cells causing restrictive cardiomyopathy and/or neuropathy. FAP renders loss of sensation in hands and feet that progress to the autonomous nervous system affecting the GI-tract, blood pressure and heart rate. Symptoms of restrictive cardiomyopathy in ATTR-CM ultimately result in heart failure.

### Localized amyloidosis

**AIAPP** - Type II diabetes is manifested by impaired glucose regulation. Long term disease is associated with dysfunctional insulin production from the pancreas. Type II diabetes is intimately linked to amyloidosis of the protein hormone IAPP in the islets of Langerhans in insulin producing beta-cells [44], the same cell that produces IAPP. The IAPP-fibrils appear to disrupt the beta-cell secretory pathway, puncture the cell and encapsulate the islet in masses of amyloid fibrils.

### Neurodegenerative amyloidosis

**Aβ and ATau -** Alzheimer’s disease is a double amyloidosis of the brain where the neuronal Aβ peptides accumulate as extracellular senile amyloid plaque and cerebrovascular amyloids and where the microtubule associated protein tau accumulates as neurofibrillary tangles inside neurons as well as in glial cells [45]. Tau fibrils are also associated with chronic traumatic encephalopathy, Progressive supranuclear palsy (PSP) and Chronic Traumatic Encephalopathy (CBD) and hereditary disorders such as Frontotemporal lobar degeneration (FTLD) and Picks disease. These amyloidogenic processes result in neurodegeneration, cognitive decline that ultimately cause dementia.

**APrP –** Prion diseases are caused by misfolding and aggregation of the prion protein (PrP) which manifests as different diseases including Creutzfeldt-Jakob disease (CJD) and hereditary diseases such as fatal familial insomnia (FFI) and Gerstmann – Sträussler – Scheinker syndrome (GSS). In prion diseases, protein aggregates, sometimes in the form of amyloid fibrils, composed of misfolded PrP which can be transmissible [46]. Prion diseases can hence be acquired through contaminated materials resulting in iatrogenic CJD and ingested through prion infected food from cattle manifesting in variant Creutzfeldt-Jakob disease (vCJD) and by cannibalism causing Kuru.

**AαSyn-** Parkinson’s disease (PD), Lewy body dementia and multiple system atrophy are associated with Alpha-synuclein (αSyn) fibril formation. In PD αSyn fibrils accumulate in and around dopaminergic neuronal cells causing depletion of the substantia nigra and loss of motor function. PD is however a systemic disease with significant CNS-gastric transfer of αsyn aggregates via the autonomous nervous system [47]. PD is multifactorial with many risk genes involved in endosomal trafficking, autophagy and mitochondrial function [48]. Aggravated risk for disease can involve many pathways.

**SOD-1, TDP43, FUS, C9orf72 –** Amytrophic lateral sclerosis (ALS) is a neurodegenerative disease causing severe motoric and autonomous neurological impairment. While ALS, nor its culprit proteins are classified as an amyloidosis or amyloid proteins the disease is a protein misfolding disorder. Sporadic disease appears associated with misfolding, aggregation and fibril formation of TDP43. Hereditary forms of ALS are associated with mutations in the genes coding for superoxide dismutase-1 (SOD-1), fused in sarcoma (FUS), and C9orf72 [49]. The latter gene results in translation of extensive dipeptide-repeat poly-peptides that readily forms fibrils. ALS is hence associated with intracellular amyloid fibril formation of either of these four proteins albeit there are many more genes implicated in this complex disease [49].

## Endoproteolysis as a mechanism for amyloidosis

Of the 42 amyloid proteins associated to amyloid diseases in humans, only 16 proteins are associated with full-length protein being deposited as amyloid fibrils [29] and 9 of these are often found co-deposited in the form of full-length protein and fragments thereof (Table 1). The overwhelming majority of amyloid proteins are hence processed. Several amyloid proteins are pre-processed during maturation *e.g*. IAPP which is processed during production of the mature IAPP protein. For many proteins it is not established if processing occurs after misfolding and aggregation, *i.e*. by a post aggregation proteolytic shaving process which may offer a selection process for efficient fibril polymorph propagation [50]. For TTR both full-length protein and C-terminal fragments of TTR (~aa 50–127) is found in ATTR deposits. The ATTR fragments have been proposed to originate from plasmin-cleaved TTR [51].

**Figure 2.**
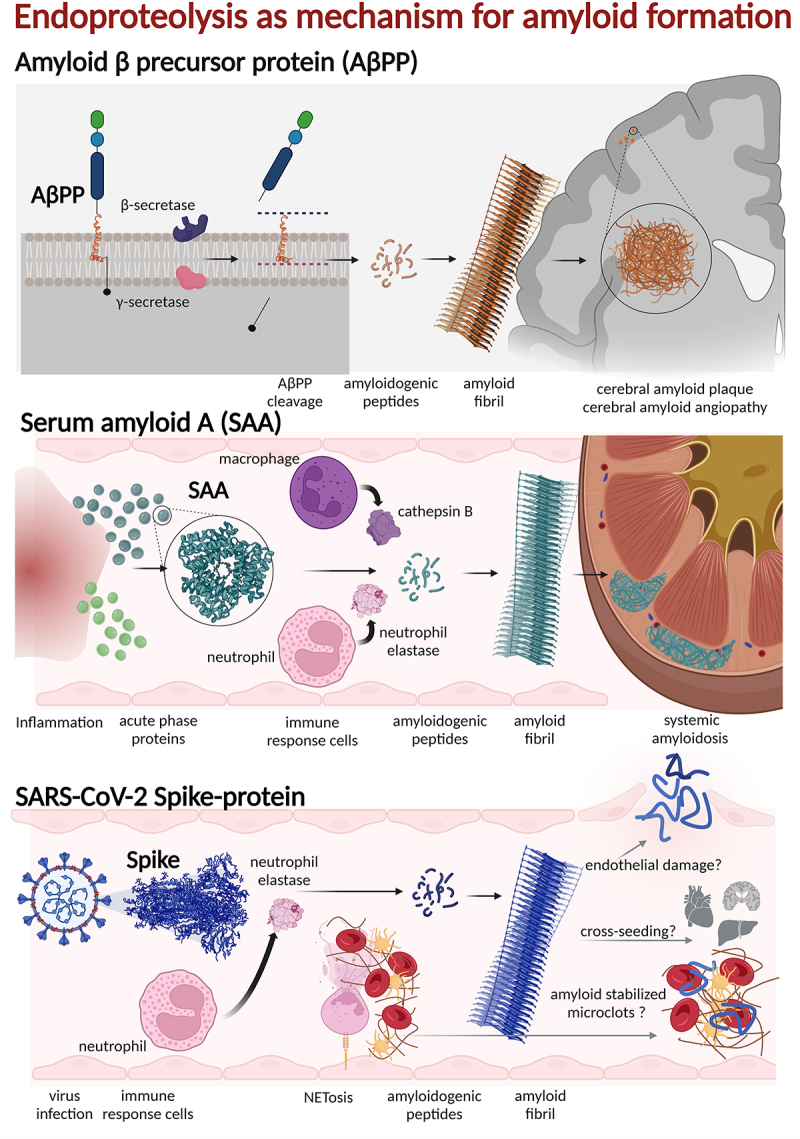
Endoproteolysis is a common and often required pathway for amyloid formation of natively folded and functional proteins.

Note: Top: The amyloidogenic Aβ peptide, a part of the trans-membrane domain of Amyloid-β precursor protein (AβPP), is cleaved by membrane bound β- and γ-secretase and exported to the extracellular space where it accumulates, deposits as amyloid fibrils and form senile plaques and CAA abundant in Alzheimer’s disease.

For a substantial number of amyloid proteins, 26 of 42, endoproteolysis of the amyloid precursor proteins is a prerequisite to produce the amyloid protein. In the absence of known genetic mutation this happens in age-associated amyloidosis for AApoAIV in cardiac and kidney amyloidosis (Apolipoprotein AIV), ATMEM106B in ageing brain, ASom in ageing pituitary (Somatostatin), AMed in ageing aortic vessel (lactadhederin), AEFEMP1 in ageing venous vessels, and ASem1 in ageing seminal vesicle (Semenogelin 1). In Alzheimer′s disease, Aβ is produced by endo-proteolysis from AβPP by BACE-1 (β-secretase) and Presenilins’ in γ-secretase in the notorious amyloidogenic pathway (Figure 2 top.). SAA1, which is overproduced during inflammation, is endoproteolysed by cathepsin B released from macrophages [52] and neutrophil elastase released from neutrophils [53] (Figure 2 middle). Furthermore endoproteolysis is a prerequisite for amyloidosis in inherited diseases for ABri and ADan in British and Danish dementia (Bri2) [54], AGel in Finnish familial amyloidosis (gelsolin) [55]. It is also likely that the endoproteolysis occurs for AFib mutations in fibrinogen amyloidosis [40] (Table 1).

Middle: Serum amyloid A (SAA) is a globular, α-helix rich acute phase protein that is highly elevated during inflammation. Inflammatory cells such as macrophages and neutrophils release proteases that cleaves the helical protein into smaller peptides, enabling it to expose amyloidogenic sequences that form amyloid fibrils found as deposits in kidney as well as other organs.

Bottom: SARS-CoV-2 Spike-protein expressed during COVID-19 infection and mRNA vaccination. When cleaved by neutrophil elastase, it will expose amyloidogenic sequences and form fibrils with the potential to inhibit fibrinolysis of formed clots and potentially cause endothelial damage and/or cross-seed amyloidogenic host protein.

## Driving forces for protein folding and avoidance of misfolding in humans

Amyloids in the mammalian context is most often, and rightfully so, considered to be detrimental alternative misfolded protein structures. Many disorders are interlinked with amyloid formation of endogenous proteins in humans and 42 proteins have to date been identified as involved in pathological amyloid formation in humans (Table 1) and several more are involved in inclusion formations [29]. Since these diseases are almost invariably lethal there is a constant evolutionary pressure driving mammalian proteins into well-performing folding machines. The folding of the polypeptide chain into a native structure that shields hydrophobic elements from interaction with the surrounding environment is in itself an effective prevention against protein aggregation and consequently, a majority of structured proteins need to be partially or fully unfolded in order to attain an amyloid structure fold [56].

Evolution spanning from prokaryotes to mammals has premiered the insertion of gatekeeper amino acids, that is, residues that have the specific role of interrupting aggregation prone regions and at the same time promote rapid and productive folding [57,58], most often these gatekeepers are prolines or charged residues. In addition it is well established that several amyloid associated diseases are tightly linked to single point mutations in the primary structure of the culprit protein and that the point mutation renders a protein that forms amyloid more readily than the wild type protein due to decreased stability of the folded state [59,60]. There are many β-sheet rich proteins in mammals and strategies by which the boundary β-strand is protected against intermolecular interaction include the formation of β-barrels and β-sandwiches, shielding of the β-strand by α-helix or loop and excessive twist of the last β-strand [61].

The mammalian genome, in contrast to viral genomes, is not sterically constrained and hence can allow for amino acid diversity between different domains within one protein as a strategy to prevent the in-register alignment of β-strands that is prevalent in β-sheet amyloid structure. The higher the amino acid diversity the lower the propensity to form amyloid where 70% or more sequence identity promotes co-aggregation whereas less than 30% sequence identity demises the probability to form aggregates [62]. Not only are the aggregating proteins themselves important players in the evolution directing away from protein aggregation and amyloid formation. A whole army of folding defenders, the chaperones and isomerases, are present in both prokaryotes and eukaryotes [57].

Chaperones acting on the unfolded polypeptide chain in close proximity to the ribosome are particularly important in the aspect of preventing amyloid formation as their mode of action is to prevent undesired interactions between nascent unstructured polypeptide chains. Although chaperones in general do not target a specific client protein but rather hydrophobic stretches or clusters of any protein there is a strong preference for interaction with polypeptide stretches that are flanked with gatekeeper residues as described above [57]. There is also evidence for co-evolution of chaperones and their client proteins in both prokaryotes [63], yeast [64] and mammalian cells [65].

An additional feature of proteostasis in humans is the notion that there is a high correlation between protein expression rate and the propensity for that protein to aggregate [66]. This appears to be an important determinant steering away from amyloid formation and is mirrored by the fact that the most common systemic amyloidoses in humans are AA amyloidosis and AL amyloidosis. These diseases are intimately coupled to severe overexpression of the culprit proteins SAA and amyloidogenic IgG light chain respectively. Proteostasis being overwhelmed is likely during ageing which is the main risk factor for all sporadic amyloidoses. Proteostasis collapse because of ageing has been demonstrated using *C. elegans* [67]. The importance of maintained protein homoeostasis to avoid age-related amyloid formation was recently reviewed [68] and further supports the conclusion that the interplay between intrinsic factors in the culprit protein (e.g. primary structure and secondary structure elements) are in an intricate and delicate balance with extrinsic factors (such as protein expression levels and molecular chaperones) in the strive to avoid amyloid formation in a dynamic system.

## Evolutionary aspects of the virus proteome

The evolutionary pressure that drives mammalian proteins away from the amyloid formation cascade does not exert the same power on viruses. Synthesis of proteins and maintenance of protein homoeostasis are energy demanding and hence costly for the protein producing organism. Therefore, all living organisms from prokaryotes to mammals benefit from maintaining a high rate of productive folding while viruses do not have to bother about the cost for protein production since someone else, namely the hijacked host cell, is paying the bill.

The prevailing strategy for viral replication is quantity rather than quality. Viruses are short-lived and the purpose of each individual viral particle seizes to exist once a new host cell is infected. The successful generation and transmission of one single virus particle will upon infection of the next host cell generate 10^2^-10^5^ or more new virions before the typical burst of the host cell in multicellular organisms [69]. Consequently, the evolutionary pressure for high productive folding rates can be assumed to be far lower than for mammalian proteins.

The size of the viral genome is however highly restricted to allow for the packing of the nucleic acid into the capsid. One strategy used by predominantly RNA viruses to expand the number of proteins that can be coded by the genome is the use of open reading frame (ORF) shift, where initiation of translation at the ribosome at the first, second or third base in the triple codon will give three different proteins from essentially the same stretch of RNA [70]. While this is beneficial to enable more proteins to be produced from the limited amount of genetic material, it might abrogate the possibility to avoid the introduction of amyloidogenic stretches in the proteome since this would require simultaneous optimization of the distinct proteins that are coded by one genome stretch.

Most viral capsids are composed of numerous identical or only a few different protein variants [71] and the prevailing folds of capsid proteins are rich in β-sheet [72]. The correct assembly of the capsid is often dependent on the presence of scaffolding proteins [73] or the viral genome which is used as folding scaffold for the proteins [71]. In a sense this can be considered an equivalent of the chaperone systems employed by all genera of life. Viral proteins produced but not assisted by these mechanisms may run rouge and start to misfold and/or misassemble.

Once the viral particle has been successfully formed it needs to be robust and sturdy to withstand the often harsh environment it needs to pass to travel between hosts. The proteins hence would benefit from being stable in its receptor binding property if that is the task or be able to interlock with fellow capsid proteins if that is the assigned function. It was recently demonstrated that there is a positive correlation between stability of the core of a globular protein and the propensity of that protein to form amyloid [74]. We hypothesize that a stable capsid or receptor-binding protein might enforce the ability to form amyloid. At the same time the viral capsid needs to be unstable enough to disassemble and unleash its nucleic acid cargo once a new host has been infected. Because of this delicate balance, virus capsids are metastable and dynamic [75] including the formation of assembly intermediates of few proteins that pack tightly together and further adhere to each other as the virion matures [76]. This means that several homologous proteins with high propensity to form β-sheet could be tightly interlinked with each other, potentially providing an excellent breeding ground for forming amyloid seed nuclei that may act as promoters of amyloid fibril formation.

There are several implications beyond those of protein stability and interaction entities of virus infection and protein production in the infected cell. High production of extrinsic proteins causes overload of the cellular proteostasis mechanisms of the host cell. The viral proteins may not have the correct gatekeeper residues that make them selected by some chaperone systems [57]. If on the other hand the chaperone system of the host cell do recognize the virus encoded proteins, there will not be enough chaperone activity in the cell to cope with both the endogenous cellular proteins and those exerted by the intruder. This overwhelming chaperone system may be a risk factor (albeit indirect) for misfolding disease. On the other hand, viruses and host organisms can co-evolve. The prokaryotic chaperone GroEL was identified as a T4-phage assistant protein for sustained infection in *E.coli*. The phage genome encodes a substituent co-chaperone, Gp31, that replaces GroES thus allowing for the bulkier virus proteins to fit into the chaperonin complex [77]. Another important global aspect of virus protein production in the context of protein aggregation and amyloid formation is that the viral proteins will not take into account the delicate balance of protein expression level and aggregation propensity that is prevailing in the host [66].

## Amyloidogenic viral proteins

In summary there are several mechanistic indications that viral proteins may be amyloidogenic. But how common is it to find virus derived proteins that form amyloid? Here we summarize some of the known virus derived amyloids coinciding with neurological long term outcomes.

**SARS-CoV-2** is a positive-sense single stranded RNA virus. It comprises four structural proteins that build up the envelope that protects the RNA and comprise several open reading frame (ORF) proteins [78]. The virus that causes COVID-19 was first described in 2019 and COVID-19 was declared a pandemic in March 2020. COVID-19 has to date (March 2024) caused more than 6.8 million registered deaths globally. The disease was first considered a respiratory disease but it was soon evident that infection caused symptoms from many parts of the body, head to toe [5] (Figure 1). The burden of long-term complications and residual symptoms after SARS-CoV-2 infection, known as long COVID or post-acute sequelae of COVID (PASC) is also heavy on society and on those who suffer from such consequences [28].

**Figure 3.**
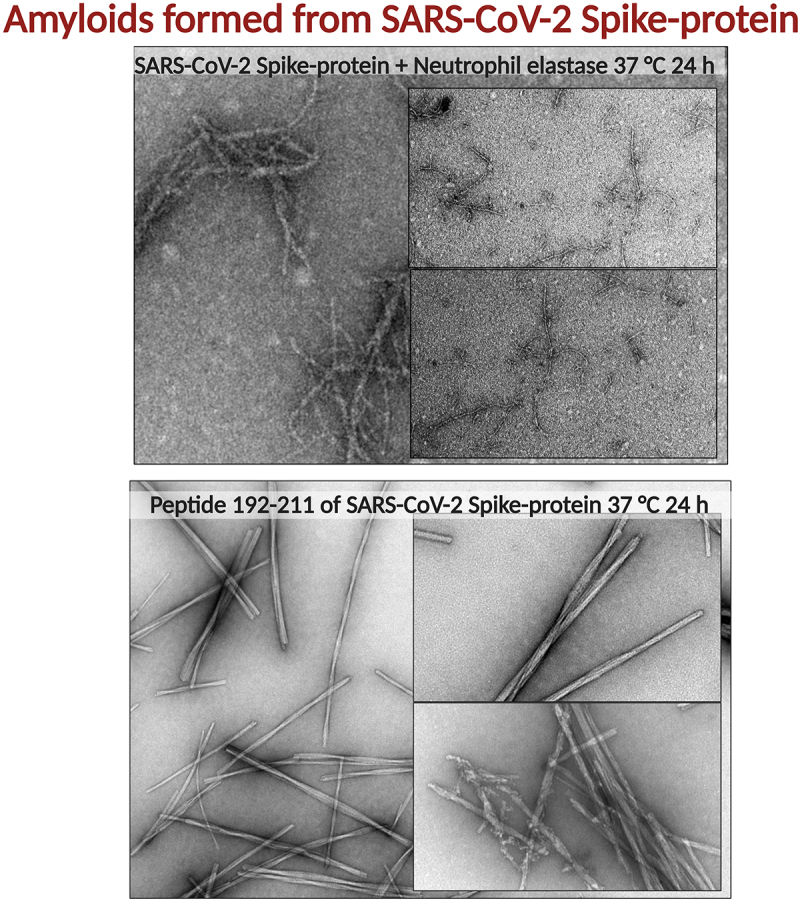
TEM micrographs of (on top) full length Spike-protein (Wuhan strain) after co-incubation with neutrophil elastase at 37°C for 24 h. The *in silico* predicted peptide 192–211 of SARS-CoV-2 Spike-protein, incubated at 37°C for 24 h. The resulting fibrils are straight, twisted and rod-like [81].

Amyloid formation of several SARS-CoV-2 proteins has been reported. Two ORF proteins, ORF6 and ORF10, were predicted to be amyloidogenic [79]. The prediction was confirmed experimentally using synthetic peptides and the resulting amyloid was toxic to the neuroblastoma cell line SH-SY5Y [79]. The nucleocapsid protein of SARS-CoV-2 and in particular the low complexity domain (that is, domain rich in glycine, serine, glutamine, and tyrosine) has been demonstrated to form amyloid fibrils and that the amyloid formation propensity is accelerated by the presence of SARS-CoV-2 RNA [80]. The SARS-CoV-2 Spike-protein (Wuhan strain) has been shown to be highly amyloidogenic. Several sequences from Spike-protein that *in silico* were predicted to be generated by elastase cleavage were also predicted to be amyloidogenic. Seven synthetic 20 amino acid long peptide sequences in full-length SARS-CoV-2 Spike-protein formed amyloid fibrils at neutral pH when incubated at 37°C [81]. Peptides from Spike-protein sequences 192–211, 601–620 and 1166–1185 were particularly amyloidogenic. Furthermore, co-incubation of full-length Spike-protein and the immune response protease neutrophil elastase resulted in proteolytic processing of the Spike-protein which then formed fibrillar clusters (Figure 3) [81]. This result indicated a plausible mechanism by which Spike-protein amyloid can be generated during infection (Figure 2, bottom) or as response to vaccination. The resulting amyloid fibrils impaired plasmin mediated fibrinolysis in an *in vitro* experiment, demonstrating that amyloid fibrils from Spike-protein may have bearing on the microclot complications seen in severe and long COVID [81] (Figure 2, bottom).

Evolution of SARS-CoV-2 has generated a cascade of novel Spike-protein variants since the beginning of 2021 [82]. Point mutations and differential processing by endoproteolysis are known to have large impact on amyloidogenicity for many human amyloidogenic proteins, *e.g*. the difference between Aβ1–40 and Aβ1–42 where the 42 amino acid long peptide is much more amyloidogenic than the 40 amino acid long alloform and an increase of Aβ1–42/Aβ1–40 ratio is connected with increased risk for Alzheimer’s disease [83]. SAA is cleaved at several sites and this generates peptides with different amyloid forming propensity [84,85]. The prion protein is highly expressed in all neuronal cells but still sporadic CJD is extremely rare. On the other hand, single point mutations in the prnp gene renders almost 100% penetrance of prion disease in the carrier [86]. Hence point mutations in the Spike-protein can potentially influence the amyloidogenicity both by altering protease recognition sites and by modulating amyloidogenic stretches in the primary sequence of the protein.

**Influenza A** is an RNA virus that is categorized into subtypes based on the two proteins present on the surface of the envelope, hemaglutinin and neuraminidase. Influenza A viruses cause seasonal flu in humans and variants of H1N1 and H1N5 gave rise to the avian flu and swine flu respectively. Pb1-F2 protein is an accessory protein expressed from the alternative+1 reading frame of the genome and is thought to contribute to elevated disease severity by increasing the inflammatory response. The recombinant expressed 90 amino acid long Pb1-F2 protein has been shown to form amyloid *in* *vitro* as well as in experimentally infected cells [87]. It was also recently shown that PB1-F2 amyloid in Influenza A infected mice correlated with proinflammatory protein signals and respiratory symptom severity [88]. Influenza A non-structured protein 1 (NS1) is a 26 kD unstructured protein that suppresses the host cell immune response and hence increase the virulence of the virus. This protein can also from amyloid *in vitro* [89].

**Zika virus** is a positive-sense RNA virus. Zika virus disease is in most cases an asymptomatic or very mild disease. However, the virus can cross the placenta if infection is active during pregnancy and cause microencephaly and other neuronal developmental issues in the foetus. It can also cause Guillian Barré syndrome, an acute autoimmune disorder that causes peripheral polyneuropathy and muscle weakness that also can affect the respiration musculature as well as the heart [90].

The proteome of the Zika virus is expressed as one single polyprotein that is threaded back and forth across the membrane of the endoplasmic reticulum (ER) and finally cleaved into several distinct proteins by host cell peptidases and viral proteases [91]. The Capsid protein is the first protein to be expressed and it is attached to the ER membrane by the N-terminal stretch called the capsid anchor (CA) comprising 18 amino acids. Synthetic CA has been shown to form cytotoxic and haemolytic amyloid fibrils in *in vitro* experiments and was suggested as one mechanistic explanation for the neuropathologic events reported for Zika virus infection [92]. Zika virus infections can have lethal neuropathological congenital effects associated with CNS development [93] but can also manifest as neurological acute and PAS in certain adults [94].

**Varciella Zoster virus (VZV)** is a double stranded DNA virus belonging to the family of Herpes viruses. Primary VZV causes chicken pox (varicella) in children. Prior to vaccine roll-out in 1995, it is estimated that 95% of the adult population had been affected by chicken pox during childhood. The virus will remain dormant in neurons in the ganglia of cranial nerves, at the dorsal root and in the autonomic nervous system. If the virus is reactivated in adults, it will cause shingles (herpes zoster). This reactivation can occur several decades after initial infection and an estimate of one third of all individuals primarily infected with VSV will encounter at least one episode of reactivation [95]. VZV reactivation is in most cases coupled to a transient yet painful malady of skin and peripheral nerves. Postherpetic Neuralgia, a state of chronic pain remaining several months after the acute symptoms of reactivation have past, is seen in 20–30% of patients and up to 2% still suffer from this after several years [96]. In Switzerland an estimated yearly incidence of 1 in 100 000 in normal population reactivation of VZV resulting in CNS infection was found. Of these, meningitis or encephalitis with severe residual symptoms or deadly outcome in almost 30% [97]. In addition, VZV can cause vascular disease that manifests as ischaemic or haemorrhagic stroke [98].

Recent data shows that VZV uses amyloid formation as a strategy to prevent the host cell from employing apoptosis as means of protecting the host organism [99]. The virus ORF20 protein RHIM forms amyloid in complex with the apoptosis signalling human ZBP1 protein and thereby blocks the apoptosis signal to reach its target.

**Herpes simplex virus 1 (HSV-1)** is another double-stranded DNA virus from the herpes virus family. The virus is very common and contagious and it is estimated that two thirds of the world population has a latent HSV-1 infection acquired during childhood [100]. HSV-1 latency is found predominantly in the trigeminal ganglia [101] and will cause episodic cold sores in the vicinity of this facial nerve typically in mucosal tissue such as mouth and lip or eye socket as result of anterograde spread from neurons to epithelial cells [102]. A more serious outcome of latent infections of herpes viruses is that they are the dominating cause of aseptic meningitis and encephalitis and especially HSV-1 is connected to encephalitis with poor outcome [103].

HSV-1 also employs the strategy of engaging parts of its proteome, the ICP6 protein, in formation of heterogeneous amyloid complexes to prevent apoptosis signalling [104]. A recent *in silico* screen of the entire proteome of HSV-1 revealed that there are additional amyloidogenic polypeptide regions encoded by the virus and that indeed a peptide derived from HSV-1 glycoprotein K formed amyloid fibrils *in vitro* [105].

**Figure 4.**
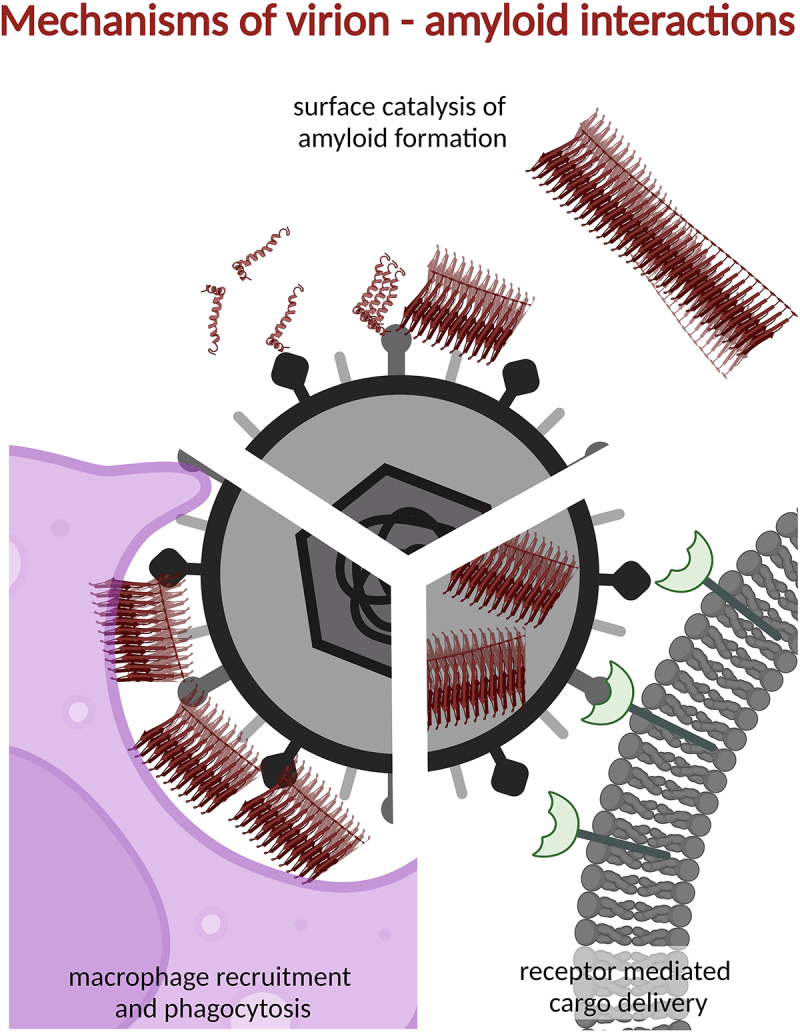
There are several mechanisms by which amyloids and viruses co-operate.

Several recent studies from research settings in Taiwan, South Korea and Sweden demonstrate an increased risk of dementia after Herpes Simplex, herpes zoster ophthalmicus or Varicella Zoster infection [106–110] with hazard ratios spanning from 1.1 to 2.4 compared to non-infected controls. The direct causal effect of the virus infection on disease progression of dementia was corroborated by the rescue effect, up to 90% lowered risk of dementia, observed in the infected group treated with anti-viral drugs at time of acute infection compared to the infected but untreated group [108–110]. HSV-1 DNA has also been found in Aβ-amyloid plaques in autopsied AD patients as well as in Aβ-amyloid plaques of non-AD controls. However, the co-localization of HSV-1 DNA with Aβ plaques was much higher in AD (72% of DNA associated to plaques) compared to non-AD (24% of DNA associated to plaques) [111]. Indeed, the HSV-1 virion surface can catalyse the amyloid formation of Aβ1–42 amyloid fibrils [112] (Figure 4). Recent data demonstrate that both HSV-1 and SARS-CoV-2 can induce amyloid formation in cerebrospinal fluid from several proteins associated to neurodegenerative diseases [113].

Amyloid formation of host encoded protein can be catalyzed on the surface of the virion [112]. Host amyloid coating the surface of the virion can activate macrophages and induce phagocytosis that in turn mediates the generation of amyloid seeds by macrophage proteolytic activity and further amyloid spreading [122]. Virus-derived vesicles (SVPs) can be loaded with amyloid content and target cell receptors leading to efficient and specific release of amyloid protein cargo to nascent host cells [131].

**Human immune deficiency virus (HIV) –** The HIV virus is a lentivirus comprising an enveloped single-stranded positive-sense RNA. The RNA will be converted to DNA upon infection and the viral genome will be incorporated in the host cell genome. HIV-associated neurocognitive disorder (HAND) is established as a secondary disorder of HIV infection, especially in HIV patients lacking adequate antiviral treatment [114]. While HAND appears different from Alzheimer’s disease, recently Aβ and HIV viral proteins gp120 [115], and most recently p17 [116] have been shown to form amyloid fibrils in brains of HAND patients. Mechanistically it is interesting to note that HIV infection is mediated using the gp120/gp41 protein system which correspond to the function of the Spike-protein of corona viruses. A fusion-inhibitor based on the C-terminal sequence of gp41, called enfuvirtide, is used clinically to prevent fusion of the HIV-virus with human cells [117]. Iatrogenic amyloid fibrils at the injection site composed of the 36-amino acid long peptide enfuvirtide was recently reported [118] (Table 1). Hence the HIV proteome contains three amyloidogenic proteins, gp120, p17 and gp41 which may have implications for the spectrum of disease phenotypes from HIV infections including HAND.

## Interactions between human amyloids and viruses

There are many mechanistic interactions between human amyloids and viral particles [119]. Some interactions are beneficial for viral entry and spread while others demonstrate protection of the potential host against the intruding virion.

### Amyloid fibrils enhance viral infection

Several proteins found in semen have been shown to dramatically increase HIV infection [120,121]. Prostatic acidic phosphatase, seminogelin-1 and seminogelin-2 are all part of the coagulum in semen and in the process they are proteolytically cleaved to form peptide fragments that form amyloid in the seminal plasma. The native function of the amyloid fibrils is yet not fully understood. However, these amyloid fibrils have been observed to attach to the surface of sperm heads and thereby triggering macrophage engulfment and activation of neutrophils. Based on this it has been suggested to be an important part in sperm selection as well as prevention of immune response to sperm in the female reproductive tract [122]. When HIV virus interacts with semen-derived amyloid to increase viral infectivity, a similar mechanism is observed. The amyloid enhances the transport of the virion across the mucosal barrier and also triggers macrophage engulfment, leading to viral infection of macrophages and also CD4+ T-cells [120]. These cells can then further mediate the infection to the novel virus recipient (Figure 4).

Similar to HIV, Ebola also has a sexually transmissible route that can give rise to male-to-female sexual transmission months after recovery from Ebola infection [123]. A pivotal role for the semen derived amyloids has also been described for these transmission events [124]

Both in the case of HIV-transmission and Ebola transmission above, the virion is assisted by the amyloid fibrils to cross epithelial membranes and enter the novel recipient. Once inside the host the virus triggers the host immune response and the virus together with the amyloid fibril is engulfed by macrophages. This mechanism not only mediates the virus infection between individuals but potentially also the spread of amyloid fibrils between organs or between cells and cell types in one organ in the same individual. Fibrils of α-synuclein have been demonstrated to enhance viral transduction into cells by concentrating the virions on the surface of amyloid particles in solutions [125]. The amyloid structure will further assist the transfection of cells using its intrinsic affinity for the cell membrane. Hence, the amyloid particles collect the virions and serve it to the cells. This is promoted by addition of chitosan, a positively charged glycosaminoglycan, or poly-Lysine, a positively charged amino-acid polymer, during fibril preparation, indication that charge can be an important parameter in the mechanism [125].

### Viruses enhance amyloid spread

The involvement of extracellular vesicles (EVs) as mediators of misfolded proteins is currently heavily researched and is an obvious candidate in the search for the route of spread of misfolded, aggregated and amyloid-structured proteins. The literature is not conclusive regarding whether the vesicles are cell type specific or not [126]. Some suggested pathways of EV mediated uptake of cargo into cells are mediated by specific host-EV surface protein dockings that appear to be highly specific [127].

Viruses and virus-derived vesicles have been shown to be efficient means of transfer of protein cargo between host cell and recipient cell [128,129]. The protein cargo is internalized inside the membrane surface of the vesicle or virion and transferred to the next cell via fusion of membranes.

Virions can in some sense be regarded as highly complex extracellular vesicles and as such could also be considered as mediators of protein cargo. However, the virion is in general tightly packed with nucleic acid, hence internalization of extra load of amyloid protein is not plausible. However, several viruses are capable of promoting subviral particles (SVPs) that essentially comprise the viral protein capsid proteins attached to membrane or lipoprotein surfaces but lack the nucleic acid [130]. In Hepatitis B infection such nucleic acid free particles are present at a ratio of as many as 100 000 SVPs to one virion and in Hepatitis C a ratio of 1 to 1 000 000 has been observed [130]. Recent studies demonstrate that extracellular vesicles exhibiting VSV glycoprotein on the surface dramatically increased the cell-to-cell transfer and infection of yeast prions and the sequence and structure unrelated mammalian scrapie prions [131] demonstrating this might be a mechanism unbiased for sequence and structure of the transferred amyloid. Additionally, EVs decorated by SARS-CoV-2 Spike-protein drastically increased transfer of the EV cargo comprising aggregated and seeding competent Tau protein [131].

Endogenous viruses, that is the presence of viral genome that has been inserted in the mammalian germline during evolution, have many similarities in protein composition to exogenous viruses that we encounter in our everyday life. Endogenous viruses are kept under close guard by several protective mechanisms [132]. However reactivation can occur and is associated to neurodegenerative and autoimmune disease as well as cancer and chronic inflammation [133]. Also such reactivated endogenous viruses have been shown to recapitulate the mediation of amyloidogenic proteins that has been demonstrated for Spike-protein and VSV-G coated EVs [134], implying that SVPs may be a general mechanism for promoting intrahost amyloid spread (Figure 4).

Neutrophils are recruited to site of infection as part of the immune response during viral infection. Neutrophil elastase will be released to disarm the intruding virions by shedding their host receptor binding proteins. However, the elastase can also generate amyloidogenic peptides from both host proteins and virus proteins. Furthermore, Neutrophil elastase possibly remaining from Neutrophil extracellular traps (NETs) have been found in amyloid deposits of several different systemic amyloidoses [135]. The amyloid per se triggers NETosis and the resulting neutrophil elastase has the capacity to digest the triggering amyloids into smaller species, generating toxic oligomers that are presumably also efficient seeds for further recruitment of proteins that are amyloidogenic, albeit still in solution [136]. Hence the system will be stuck in a vicious circle.

## COVID-19 sequelae with similarities and possible connections to virus derived amyloid

### Systemic amyloidosis

A cytokine storm during COVID-19 infection will render SAA1 being severely upregulated rendering risk for AA amyloidosis [137]. A recent paper reported on a case of suspected COVID-19 acceleration of a rare dual AL and AA amyloidosis with notable hepatic deposition. The SAA1 i.e. the AA precursor protein is synthesized in the liver and could be triggered by COVID-19 infection and associated with AL-amyloid seeds [138]. This case would strengthen the hypothesis of AA amyloidosis is a factor causing systemic complications after COVID-19 disease [139].

Transthyretin cardiac amyloidosis, ATTR-CM, was discovered in two studies of post-mortem patients after COVID-19 and either the disease appeared worsened by the infection or was more prevalent in COVID-19 deaths [140,141]. In one patient myocardial inflammation was concomitant in with ATTR-CM [141]. ATTR-CM is common in elderly patients making it likely that the disease outcome could be worsened. Deposition of ATTR occurs systemically but is most prevalent in peripheral nerves for familial disease and in the heart for wild type amyloidosis. The latter is a significant issue for ACE2 mediated SARS-CoV-2 Spike-protein association as ACE2 expression is confined to the endothelial cells of the arteries, arterioles, and venules of the heart and kidney to regulate Renin-Angiotensin I-II conversion rendering high blood pressure a risk factor for COVID-19 death. ATTR accumulates surrounding the cardiomyocytes depriving the heart from normal function. Vascular association of SARS-CoV-2 infection is likely detrimental and a possible worsening of ongoing ATTR amyloidosis [142]. Systemic amyloidosis patients (AL and ATTR) showed excess mortality when suffering from COVID-19 compared to otherwise healthy individuals [143].

#### Type II diabetes

There is highly elevated risk for Type II diabetes after COVID-19 infection [144]. Recent data support invasion of SARS-CoV-2 in exocrine pancreatic cells in autopsied patients [145]. Pancreatic localized IAPP amyloidosis known to be associated with type II diabetes offers a putative link between beta-cell proteostatic challenge due to SARS-CoV-2 replication or cross-seeding of IAPP from SARS-CoV-2 amyloidogenic proteins which could exacerbate IAPP amyloidosis and elevated incidence of the disease.

#### Neurological diseases

A recent meta-data mining study found correlations between 45 viral exposures with elevated risk for Alzheimer’s disease (AD), amyotrophic lateral sclerosis (ALS), generalized dementia, vascular dementia, Parkinson’s disease (PD), and multiple sclerosis (MS) [2]. A wide variety of neurological disease symptoms have been described associated with post COVID-19 infection [146]. The risk ratio was significantly elevated for memory impairment and AD, peripheral neuropathy and paraesthesia; episodic disorders (migrane, epileptic episodes), movement disorders (abnormal movements); mental health disorders (depression, anxiety and stress); sensory disorders (hearing loss, vision impairment); neurological impairment (dizziness). Many of the above neurological disorders can be associated with microclots or vascular impairment due to cerebrovascular disorders (ischaemic stroke and tia) being elevated. We briefly summarize some reported associations below.

#### Alzheimer’s disease

In a rather unique study comparing brain MRI of people pre-pandemic and after exposure of early strains of SARS-CoV-2 detected elevated cognitive decline, degeneration and brain atrophy in COVID-19 patients compared to controls [147]. In other studies, Aβ aggregates were found in young patient brains from COVID-19 infected patients [148]. These studies and several more have associated COVID-19 with New-Onset Alzheimer’s Disease [149]. Cross-seeding of amyloidogenic SARS-CoV-2 proteins and Aβ and tau are mechanisms worth to consider as an explanation for this correlation. Herpes reactivation in COVID-19 also merits suspicion in that the severe effects of herpes reactivation causing aseptic meningitis and encephalitis [103] as well as increased risk of dementia [106–110] may come into play.

#### Creutzfeldt-Jakob disease

Creutzfeldt-Jakob disease is exceptionally rare. It affects 1–2 people per million per year worldwide [150]. It is therefore rather surprising that there are several case reports that connect Creutzfeldt-Jakob disease with COVID-19 infection [151–155]. The mechanism is currently not known but cross-seeding of amyloidogenic SARS-CoV-2 proteins and PrP is a possible mechanism for this putative association.

#### Parkinson’s disease

Parkinsonism has been reported as a significant PASC symptom and suggested increased incidence of PD after COVID-19 infection [156]. While the mechanistic connection is not established there are putative links from a pathology perspective. A post-mortem case report presented a 76 years old PD patient with prolific SARS-CoV-2 Spike-protein aggregates in brain and the heart [157]. No evidence for N-protein was detected and these aggregates were therefore ascribed to protein products from mRNA vaccination coding for Spike-protein production. The patient rapidly deteriorated following vaccination. Cross-seeding of amyloidogenic SARS-CoV-2 proteins and αsyn and tau are potential mechanisms to explain this observation.

#### Amytrophic lateral sclerosis

For ALS there has been reports of accelerated disease after COVID-19 infection [158]. The mechanism is currently not known but as in the previous examples cross-seeding of amyloidogenic SARS-CoV-2 proteins with SOD-1, TDP43, FUS, C9orf72 proteins can be considered in addition to proteostasis challenge and cellular trafficking disruption.

#### Vascular and cerebrovascular amyloidosis

Cerebral amyloid angiopathy (CAA) is a prevalent pathology in amyloidosis. Eight amyloid proteins, Aβ, ATTR, ADan, ABri, ACys, APrP, AGel, AMed [159,160] (Table 1), are known to form amyloid in CAA and can cause vascular dementia. These vascular deposits are associated with accumulation of ApoE, Serum amyloid p component (SAP) and glycosaminoglycans (GAGs) which are amyloid associated proteins and glycans [161] which together severely impairs the vasculature increasing the risk for haemorrhagic stroke. Hence COVID-19 with associated risk for vascular pathology may worsen the risk for vascular damage in a CAA affected patient.

## Amyloidogenesis, abnormal blood clotting and fibrinolysis in COVID-19

Disorders discussed throughout this review are driven by complicated overlapping mechanisms. Haemostasis, amyloidogenesis, and SARS-CoV-2 viral infection conceptually merge how viral and human amyloids facilitated by a carnival of endoproteolytic processes can affect human health.

**Figure 5.**
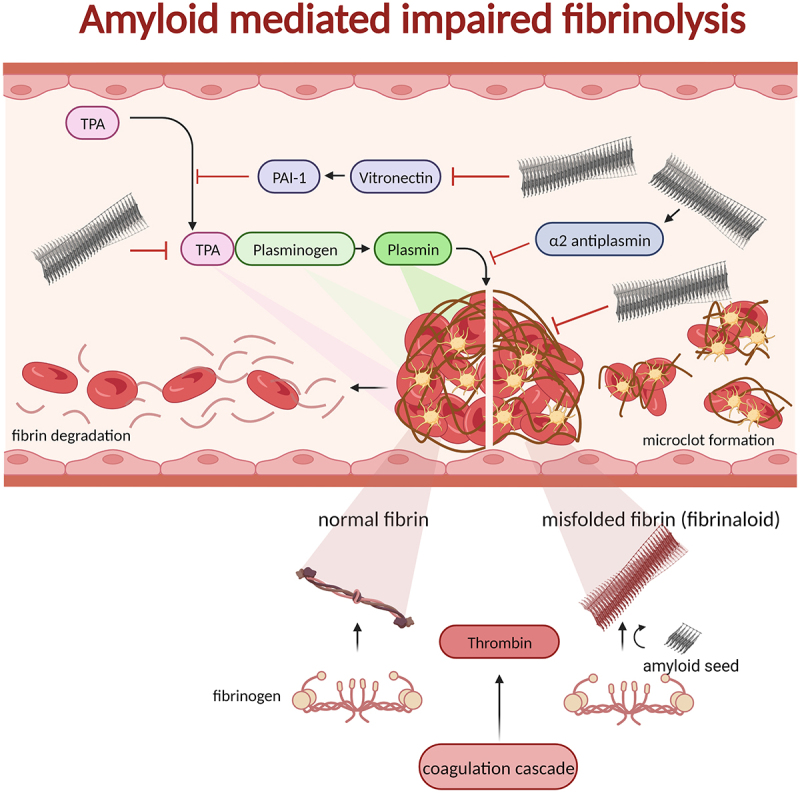
Blood clots formed by thrombin processing of fibrinogen to fibrin recruiting blood platelets in a polymerizing fibrous web. The process of fibrinolysis is regulated by several proteins ultimately affording active plasmin as the proteolytic enzyme to digest insoluble fibrin and, hence resolving the clot. The normal process is shown on the left side of the clot in the figure. If fibrin on the other hand is generated from fibrinogen by thrombin (possibly even without thrombin) in the presence of amyloid seeds the fibrin can form misfolded fibrin (fibrinaloid) which cannot be digested by plasmin (right side of the figure). In addition to seeding misfolding of fibrin, amyloid fibrils are inactivating and activating proteins involved in fibrinolysis: tPA, vitronectin, α2-antiplasmin, and plasmin. Hence, there are many associations between fibrinolysis and amyloid disease as discussed in the main text.

The association of blood clotting disorders in COVID-19 patients is established. The majority (>90%) of COVID-19 patients in an IUC multicenter study very early in the pandemic, suffered from different coagulation abnormalities and a high number of the patients developed life threatening complications due to thrombosis [162]. The symptoms from long-COVID/PASC are also closely linked to fibrinolytic disturbances with persistent amyloid-like fibrin thrombi [163]. Blood coagulation and fibrinolytic processes are regulated by proteolytic processing and is affected by viral infections. Likewise, amyloidogenic protein production as discussed above, is mediated by a vast range of endoproteolytic events. The key protein in coagulation processes is fibrin(ogen). Fibrinogen is composed of three separate protein subunits making heterotrimers that dimerize into a hexameric oligomer (Figure 5 bottom). Fibrinogen is synthesized in the liver at a very high rate 1-5 g/day rendering a high concentration (3 mg/ml) in blood plasma. This high expression of fibrinogen is further elevated during inflammation and in COVID-19 infection [164].

For coagulation and fibrin formation to be initiated thrombin cleavage of pro-protein processing occurs by cleavage of fibrinogen alpha and beta chains to convert fibrinogen to insoluble fibrin. Fibrin is thereafter cross-linked by FXIII by covalent cross-linking to stabilize the clots [40]. Fibrin is degraded by a process called fibrinolysis. Fibrinolysis is initiated by tPA-binding to fibrin which activates plasmin from the precursor plasminogen locally to degrade the fibrin clot (Figure 5, left).

Work on systemic amyloidoses have linked acquired blood coagulation and fibrinolysis disorders with amyloid disease [165]. AA and AL amyloidosis were associated with acquired blood coagulation deficiency by lack of activated FXa [166], von Willenbrand factor (vWF) [167], and factor V (FV) [168]. In addition to the acquired deficiencies that can manifest as coagulation disorders, it was implicated that the fibrinolytic system was activated in amyloidosis. AL and AA amyloidosis patients had elevated levels of circulating plasmin-α2-anti-plasmin (PAP) and increased plasmin generation indicating activated fibrinolysis [169]. The biological role for activation of the fibrinolytic system in amyloid disease was hypothesized to work as a protective system for removal of misfolded protein aggregates including amyloids [42]. It has been shown that tissue-type plasminogen activator (tPA) is activated through the interaction with amyloid structures. This included synthetic amyloid fibrils of a fibrin/ogen-peptide and Aβ resulting in plasminogen cleavage and release of active plasmin *in vitro* [42]. This would render free plasmin to digest aggregated misfolded proteins. Taken together these data suggested less efficient coagulation and increased fibrinolysis in systemic amyloidosis [165]. Iatrogenic tPA-mediated severe haemorrhagic strokes was recently implicated as a combinatorial side effect of the novel Aβ-protofibril targeting Lecanemab antibody [170]. The progression and the extent of the haemorrhage of the afflicted patient were rapid. The adverse clinical trial death was possibly associated with tPA over activating plasmin formation locally by Aβ-CAA engagement of tPA. The mechanism of tPA binding to amyloid fibrils could in patients with normal tPA levels render the fibrinolytic system impaired. Competition of tPA captured on amyloid fibrils rather than on fibrin can result in active plasmin mislocalization. In COVID-19 fibrinolysis appears to be impaired in its capacity of plasmin degradation of amyloidotic fibrin clots [163]. Misfolded plasmin-resistant fibrin in COVID-19 plasma is referred to as fibrin amyloid or fibrinaloid [163]. It was observed in COVID-19 patients that plasminogen activator inhibitor-1 (PAI-1), vitronectin, plasminogen, and tPA were elevated and fibrin clots formed from COVID-19 patient plasma showed altered fibrin network structures with elevated cross-linking and shorter fibrin fibres [164].

Vitronectin is also known as plasminogen activator inhibitor-1 (PAI-1) binding protein and has a functional role in the innate immune system and maintenance of thrombosis and fibrinolysis [171]. Vitronectin is proteolytically degraded by thrombin, elastase and plasmin. Proteolysis reduces its ability to stabilize PAI-1. Hence its degradation pathway switches between being antifibrinolytic (bind PAI-1 and stabilize its inhibitory role) and profibrinolytic (promoting plasminogen activation). Vitronectin is a common protein associated with amyloid deposits [172]. Impairment of vitronectin pathways could exacerbate a fibrin-clotting process. Vimentin, just as vitronectin, is a common component of amyloid fibril deposits in amyloidosis [40]. Vimentin has been identified as an attachment factor for SARS-CoV-2 entry to endothelial cells [173]. This association was shown to be directed by the SARS-CoV-2 Spike-protein implicating direct protein-protein interactions on the path towards amyloid formation.

Fibrin is also involved in atherosclerotic plaque formation. Whereas no wild type fibrin sequence has been found in familial AFib fibrin-amyloids, in atheroscleorosis [174] amyloid deposition is a common component of atherosclerotic plaques where the main amyloid component is composed of wild type ApoA-I amyloid fibrils [175]. Atherosclerotic plaque is a complex structure of lipids co-aggregated with many amyloidogenic proteins: fibrinogen, transthyretin, ApoA-IV [176], and amyloid associated proteins serum amyloid P component (SAP) and vimentin [177]. This strengthens the hypothesis that fibrin(ogen) is an aggregation prone protein and a key player regarding its abundance of substrate for formation of pathological clogged vasculature in conjuncture with apo-lipoproteins that are notorious for amyloid deposition (Table 1) [178]. There are proteomic overlaps between atherosclerotic plaque and fibrinaloid microclots found in COVID-19 patients, *e.g*. Apolipoprotein C-II, ApoA-II, SAA, are amyloidogenic components in the microclots [179]. Insoluble microclot compositions from COVID-19 patients have been further shown to comprise Coagulation Factor XIII, plasminogen, fibrinogen alpha-chain, a2-antiplasmin, von Willebrand Factor, C-reactive protein, SAA-4 and complement component C7 [180]. Taken together all these vicious liaisons suggest that SARS-COV-2 Spike-protein in combination with inflammation, impairment of blood-coagulation and fibrinolytic systems compose the perfect environment for amyloid and protein aggregation formation (Figure 5). This process may be the associated with thrombotic events reported in COVID-19 patients [142].

## Concluding remarks

As we have outlined in this review there are many phenotypic, biologic, and molecular overlaps between viral infections and amyloidosis. The proteopathic overlaps are concerning and are reminiscent of the early discussions on the strange nature of prions as infectious agents that goes back to the Gajdusek Nobel Prize in 1976. The Nobel committee press-release stated: ‘The possibility that further diseases in the brain e g different forms of presenile dementia, Parkinson’s disease, amyotrophic lateral sclerosis and multiple sclerosis possibly may be caused by infectious agents is the subject of continuous studies … . Even if much knowledge remains to accumulate concerning slow infections in the brain of the type described it is clear today that these infections are caused by agents of a completely new type, which initiate a pathologic process of hitherto unknown kind. This implies that the definitions of diseases, which may potentially be of infectious origin have to be markedly widened’ [181].

Never before have so many people been subjected to a totally novel virus in such short period of time as during the ongoing COVID-19 pandemic. The current situation, in wake of the SARS-CoV-2 virus, makes us wary of the amyloidogenesis of SARS-CoV-2 proteins, especially the Spike-protein [81]. Following the developments of clinical features during the COVID-19 pandemic and reviewing the literature of interactions between viruses in general, and SARS-CoV-2 in particular, we conclude it highly plausible that viruses play a role in the development several amyloid related diseases. In many instances with several years spanning between active viral infection and disease presentation.

We also emphasize that amyloidogenic routes may play a vital role in long COVID/PASC as well as debilitating vaccine side effects [182,183], hence mandating an awareness for those in contact with patients as well as basic scientists with interest in protein misfolding and amyloid formation. We cannot yet grasp the long-term consequences of the pandemic since too short time has passed. However, we can enhance the efforts of developing anti-viral treatments and, in parallel, molecular diagnostics and treatment protocols for those suffering from these conditions. The phenotypic overlaps between COVID-19, PASC, and undesired effects of Spike-protein based vaccines with those found for systemic amyloidosis, cardiac and neurologic disease may have a common molecular backdrop and mandates further investigation in the intersection between virology and amyloid research.
